# A polymer of calcium aluminate and water glass as cement substitute

**DOI:** 10.1038/s41598-026-50294-8

**Published:** 2026-05-02

**Authors:** Bernd Spangenberg, Jan D. Epping

**Affiliations:** 1 Department of Process Engineering, University of Offenburg, Badstraße 24, 77652 Offenburg, Germany; 2 Fakultät II: Mathematik and Naturwissenschaften, Institut für Chemie, School of Mathematics and Natural Sciences, Department of Chemistry, Technische Universität Berlin, Straße des 17. Juni 135, 10623 Berlin, Germany

**Keywords:** Geopolymer binder, Alternative to ordinary Portland cement, Calcium aluminate cement, Water glass, Concrete from dune sand, Chemistry, Materials science

## Abstract

Calcium aluminate cement (CAC) is a suitable source of tetrahedral aluminum, which reacts with tetrahedral silicon from water glass (aqueous sodium or potassium silicate solution) in an exothermic reaction, forming inorganic polymer structures that are stable at high temperatures. All tetrahedrally configurated aluminum atoms of the various calcium aluminates tested, react with tetrahedral silicon groups to form –O–Si–O–Al–O– chains with an average Si/Al ratio near of 1. Octahedrally configurated aluminum, as in γ-Al_2_O_3_, does not react at room temperature. The sodium-to-silicate ratio of the silicate solution was measured to be 1:1, so most water glasses require activation with NaOH. The number of positive charges in the activated water glass (Na^+^ or K^+^ ions) corresponds to the negatively charged Al^IV^ centers of the CACs, suggesting a sodium-to-aluminum ratio of 1:1. The polymer hardens at temperatures between 4 and 65 °C. The workability is identical to that of ordinary Portland cement (OPC) and enables concrete structures to be cast at room temperature without additional energy supply. The new polymer has the potential to completely replace OPC in all its applications, but can reduce annual CO_2_ emissions from OPC production worldwide from the current 8% to less than 2%.

**Supplementary Information:**

The online version contains supplementary material available at 10.1038/s41598-026-50294-8.

## Introduction

We present an inorganic, high-temperature stable polymer in which silicon-oxygen tetrahedra and negatively charged aluminum-oxygen tetrahedra form macromolecules without carbon atoms, that has the potential to change the construction industry. Today, ordinary Portland cement (OPC) is the construction material of choice because it is easy to handle and well suited for casting stone structures. Every ton of OPC generates at least half a ton of CO_2_^1^. In 2016, the OPC industry made a significant contribution of 8% (approximately 3 GT) to global CO_2_ emissions^1,2^, with about 60% of the CO_2_ emissions being released during the calcination (decarbonization) of limestone^3^. A critical analysis to OPC alternatives suggests that there are few cement systems that would deserve serious attention in terms of global reduction of concrete-related CO_2_ emissions, but to date no breakthrough has been achieved^4^. Therefore, new low-CO_2_ or CO_2_-free binder systems are being sought worldwide^4^, and alkali-activated cements are becoming the focus of interest^5–8^.

The history of alkaline cement binder began in 1908 with a patent by H. Kühl^9^. These binders, also known as geopolymers, mineral polymers or soil cements, are obtained from granulated blast furnace slag, fly ash and metakaolin. Currently, very little commercial use is made of these materials. On the resource side, there are major limitations as blast furnace slag and fly ash from coal combustion are still used as blends in the OPC industry and it is not certain that they will be available in the future due to the 2015 Paris Agreement. In addition to slags and fly ash, small quantities of clays and natural pozzolans are currently used as substitutes in the OPC industry. Natural pozzolans are abundant but locally limited^3^. The alkaline activation of natural pozzolans is known for more than 2000 years^10^. Roman concrete, *Opus Caementitium*, was used extensively in the Roman Empire from the 1st century BC onwards. Publications show that tetrahedral aluminum is essential for the stability of Roman concrete^11–14^.

From our experience with alkaline-activated water glass (aqueous sodium silicate solution), we know that mostly calcium-containing mixtures harden immediately at room temperature, so we have focused on reactions with calcium aluminates^15^. Surprisingly, there are only a few publications describing such a reaction^16–20^. Several modifications of calcium aluminates are known, but we have only used monocalcium aluminate CaO*Al_2_O_3_ (CA) and monocalcium dialuminate CaO*2Al_2_O_3_ (CA_2_) besides dodecacalcium hepta-aluminate, 12CaO·7Al_2_O_3_ (C_12_A_7_), which are cheaply available in calcium aluminate cements (CACs)^15,21^. CACs harden when mixed with water to form hydrated calcium aluminates with an octahedral structure^22^. We are convinced that a combination of activated water glass, sand^23^ and calcium aluminate is a suitable substitute for OPC in terms of availability, processability and stability, and we believe that this combination can solve most OPC environmental problems.

## Experimental results

At room temperature, CACs do not react with a typical water glass of modulus 3.4 (35.8 g sodium silicate dissolved in water to obtain 100 g water glass), although CACs rapidly form hydrates with water and harden^22^. However, when a suitable amount of sodium or potassium hydroxide is added, the viscosity of the mixture nearly instantly increases and after a few hours the suspension solidifies. Water glasses with a modulus of 1 or 2 do not require alkaline activation and react readily with CACs. A similar behavior is known from geopolymerization reactions. Singh et al. found that in a mixture of water glass and metakaolin without addition of NaOH “the hydrolyzed aluminate species formation is very slow and polycondensation reactions for geopolymerization are incomplete”^24^.

### Infra-red measurements of the reaction mixture

**Fig. 1:**
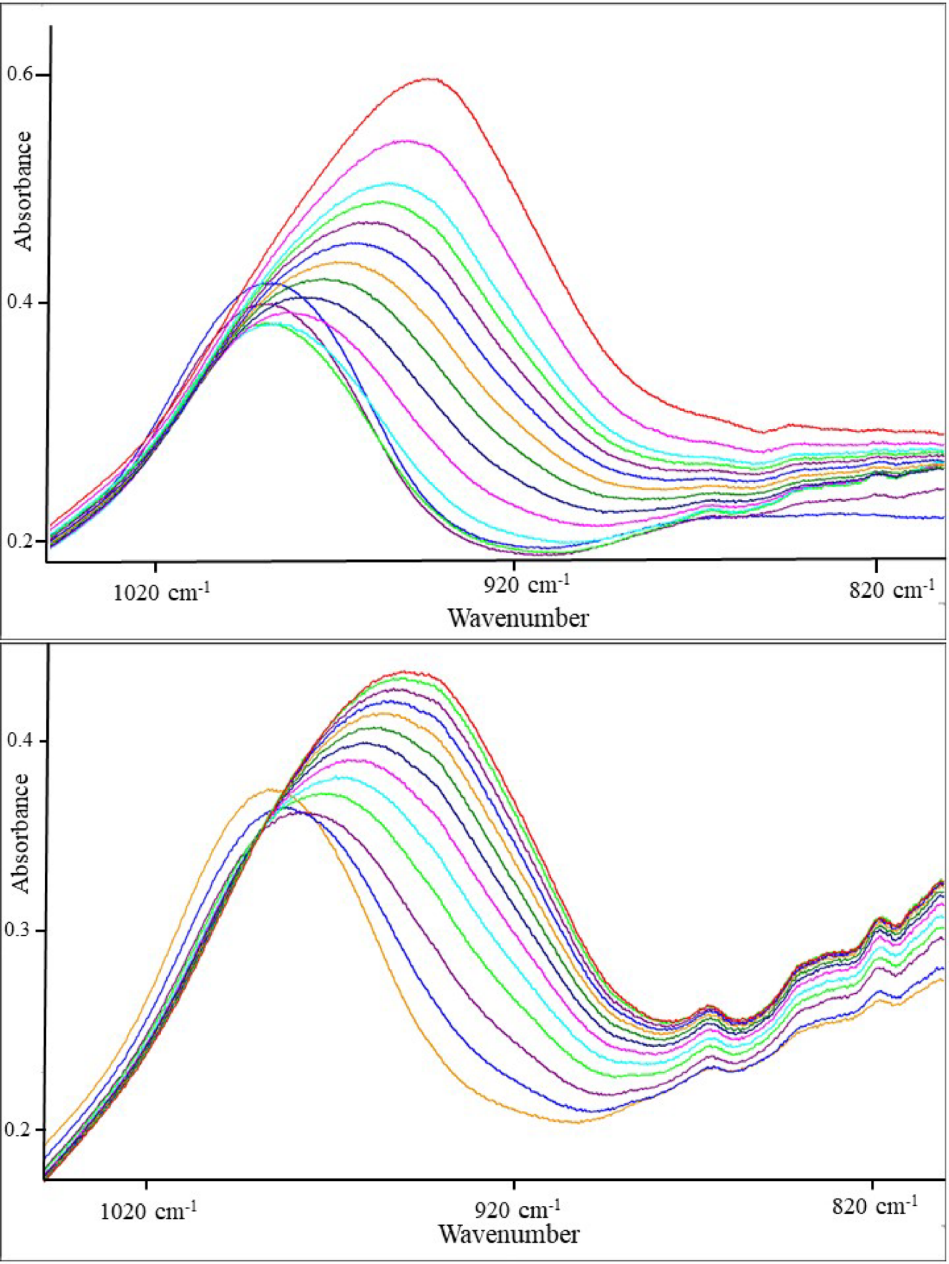
Time-dependent IR spectra of the reaction. Top: A mixture of water glass (Na38/40), NaOH and CAC (CA-14 M) with a Si/Al-ratio of 7/10 solidified within 210 min (measured in 10 min intervals). Bottom: The same compounds with a ratio of Si/Al = 7/30 solidified within 60 min (measured in 5 min intervals). The NaOH concentration in the water glass is 9% (w/w).

Attenuated total reflectance Fourier transform infrared spectroscopy (ATR-FTIR) can be used to determine whether covalent bonds were formed during a reaction. Figure 1 (top) shows the change of the IR absorption signal at 985 cm^−1^ in a mixture of 9% (w/w) NaOH in water glass modulus 3.4 with calcium aluminate cement (CA-14 M) in the ratio of Si/Al = 7/10. Within 210 min, the absorption maximum of the water glass changes from 985 cm^−1^ to 940 cm^−1^. The shift toward lower wavenumbers with increasing proportions of Al atoms in the system suggests a polymerization reaction in which a network of –Si–O–Si– and –Si–O–Al– bonds is formed^25–28^. The substitution of Si in a –Si–O–Si group with Al leads to a reduction in the bond angle with oxygen and thus to the appearance of the signal at a lower wavenumber, which is due to the weaker bond strength and the fact that the Al–O bond is longer than the Si–O bond. The results suggest that a silico-aluminate is formed as the reaction product^28^.

Figure 1 (bottom) shows the change in the IR absorption signal for a Si/Al = 7/30 mixture over 60 min. Characteristic –Al–O–Al– bands of calcium aluminate can be seen at 866, 833 and 817 cm^−1^, indicating an excess of unreacted calcium aluminate. The concentration of CAC in the reaction mixture influences the hardening time. The more CAC present, the faster the reaction. Very high CAC concentrations result in solidification after a few minutes.

These experiments show that the alkalinity of the water glass and the concentration of CAC influence the reaction. The objective of this work is to find out which kind of calcium aluminates react to determine the optimum silicon-to-aluminum ratio and, due to the activation of water glass by NaOH, the optimum sodium-to-silicon ratio (the water glass modulus).

### CAC amount optimization by compressive strength measurements

**Fig. 2:**
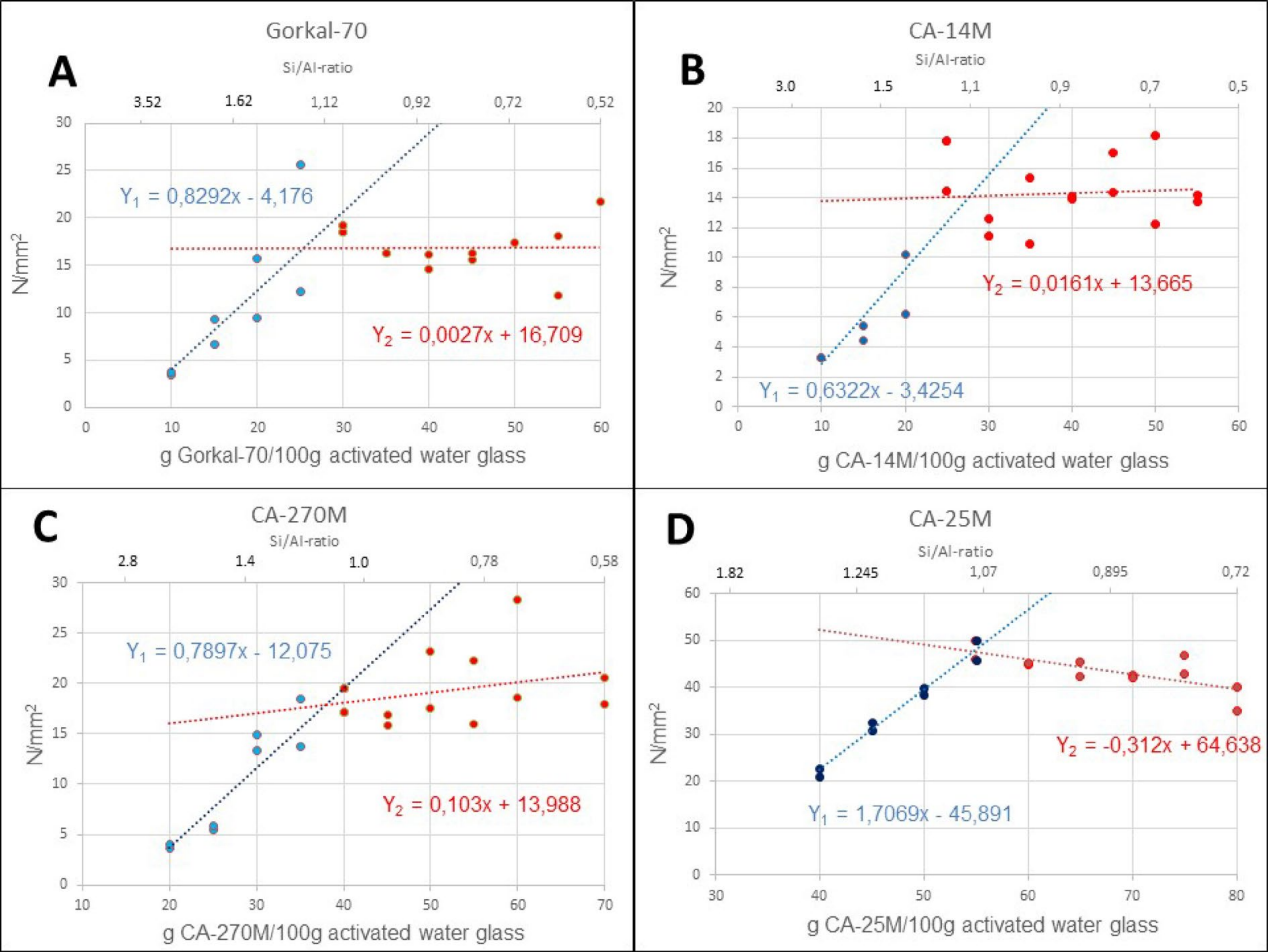
Compressive strength values in N/mm^2^ of samples made from 100 g activated water glass (activated with 8.8% NaOH), plotted against different amounts of CACs brought to reaction.

To determine the optimum amount of CAC for activated water glass, four different CACs with different contents^29,30^ of Al_2_O_3_ (A) and the most important calcium aluminates CA, CA_2_ and C_12_A_7_ were selected for the investigations. They cover the entire available spectrum of high alumina containing CACs (Table 1). To characterize these CACs, we gave them a stoichiometric formula and a hypothetical molecular mass (*M*^*CAC*^) that does not deviate by more than 1.8% from their real composition. The compressive strengths of mixtures with 91.2 g water glass (modulus 3.4, containing 35.8% sodium silicate) and 8.8 g NaOH for activation, sand and various proportions of CACs were then measured. In Fig. 2, the compressive strength is plotted against the different amounts of CACs.

**Table 1:** Composition, stoichiometric formula and molecular weight of various CACs with the abbreviation C=CaO and A = Al_2_O_3_.

Name	CA (%)	CA_2_ (%)	C_12_A_7_ (%)	A (%)	Stoichiometric formula	M^CAC^ (g/mol)	Al + Ca-atoms (in CACs/mol)	
Gorkal-70^30^	76.3	22.8	–	–	11CA*2CA_2_	2258	30	13
CA-14M^29^	59	39	1	1	5CA*2CA_2_	1310	18	7
CA-270^30^	57.9	17.8	–	24.3	5CA*CA_2_*3A	1356	14	6
CA-25M^30^	40.7	10.2	4.9	44.2	72CA*11CA_2_*C_12_A_7_*122A	28,067	202	95

The last two columns show the number of aluminum (*k*^*A*l^) and calcium atoms (*k*^*Ca*^) in the stoichiometric formula.

In the experiments, all CACs in Fig. 2 show the same behavior. With increasing addition of CAC, the compressive strength of the cured sample also increases. Above a certain amount of CAC, the compressive strength remains constant, so the curve can be approximated by two linear x-depending functions, resulting in a straight line with increasing values (Y_1_) followed by a straight line with nearly constant values (Y_2_). If both Y values are set equal and resolved to x, the value at which both functions meet can be calculated. The intersection point describes the inflection point of the entire diagram, which is considered the optimal amount of CAC, since larger CAC amounts contribute nothing or almost nothing to the final compressive strength. This optimal amount of CAC is 26.03 g for Gorkal-70, 27.74 g for CA-14 M, 37.95 g for CA-270 M and 54.75 g for CA-25 M (please see Table 2).

The experimental Si/Al ratios of the optimal amounts—calculated using only the aluminum content of the calcium aluminate—are 1.21 for Gorkal, 1.09 for CA-14 M, 1.06 for CA-270 M, and 1.06 for CA-25 M. In Fig. 2, the corresponding Si/Al ratios are plotted on the upper x-axis, with values close to 1 observed at the optimal CAC amount. The average Si/Al ratio for all four CACs, calculated without aluminum atoms from Al_2_O_3_, is 1.11, and 1.07 when the value for Gorkal-70 is not taken into account.

**Table 2:** Calculation of the optimum amounts of CAC when reacting with water glass, depending on whether the different types of calcium aluminate (and aluminum oxide) react or not.

Name	CA	CA +CA_2_	CA+CA_2_ +C_12_A_7_	All+A	Optimal CAC amount
Gorkal-70^30^	42.8 g (1.64)	**31.4 g (1.21)**	–	–	**26.03 g**
CA-14M^29^	54.6 g (1.97)	**30.3 g (1.09)**	–	–	**27.74 g**
CA-270^30^	56.5 g (1.49)	**40.4 g (1.06)**	–	28.3 g (0.74)	**37.95 g**
CA-25M^30^	81.2 g (1.48)	62.2 g (1.14)	**57.9 g (1.06)**	26.2 g (0.48)	**54.75 g**

The Si/Al ratios given in brackets were calculated based on the silicon content in the water glass and the experimentally determined CAC amounts, depending on whether the different types of calcium aluminate (and aluminum oxide) react or not. The last column shows the experimentally determined optimal CAC amounts for the reaction with water glass for comparison with the best fitting CAC reaction behavior (bold).

The value of the modulus defines the molar mass of the water glass. For a modulus of 3.4, the water glass formula is Na_2_O*3.4SiO_2_ with a molar mass of *M*^*WG*^ = 266.3 g/mol. The use of 91.2 g water glass (35.8 g sodium silicate in 100 g water glass) in the mixture results in a silicon molarity of 91.2 g*0.358 g/g*3.4/(266.3 g/mol) = 0.41686 mol. To calculate the required amount of CAC, the silicon molarity must be multiplied by the molar weight of the CAC and divided by the number of reactive aluminum atoms. As an example, assuming that only CA reacts in Gorkal-70 (11CA*2CA_2_), then we have 22 reactive Al atoms in the formula (A = Al_2_O_3_), which leads to an amount of 0.41686 mol*(2259 g/mol)/22 = 42.8 g Gorkal-70. This is calculated in Table 2 for all the different aluminum atoms of the various calcium aluminates in the CACs and also for Al_2_O_3_. The best agreement between these calculations and the experimental data is marked in bold and shows that the aluminum atoms of the calcium aluminates CA, CA_2_ and C_12_A_7_ react. The experimental data also show that, taking only the Al atoms of the calcium aluminates into account, the Si/Al ratio for all calcium aluminate cements is close to one in the optimum (see Table 2). The aluminum-atoms of Al_2_O_3_ show no reaction with water glass at ambient temperature.

### Optimization of the Si/Al ratio by ^27^Al-MAS-NMR measurements

Nuclear magnetic resonance (NMR) is a versatile method for measuring all nuclei with non-zero nuclear spin, such as ^27^Al or ^29^Si. Magic angle spinning (MAS) allows NMR measurements on solid samples with increased spectral resolution. ^27^Al-MAS-NMR can distinguish between sixfold coordinated Al-centers (Al^VI^) showing signals in the range of 0–20 ppm, fivefold coordinated Al^V^ showing signals in the range of 20–50 ppm, and fourfold coordinated Al^IV^ with signals in the range of 20–90 ppm^31^. ^27^Al is a so-called quadrupolar nucleus (with nuclear spin *I > 1/2*). These quadrupolar nuclei are particularly sensitive to electric field gradients in their surroundings and therefore show a significant line broadening in asymmetric surroundings of the ^27^Al nucleus. In its ^27^Al-MAS-NMR spectrum, monocalcium aluminate (CA) shows a signal at 81 ppm, which can be attributed to Al^IV^ coordinated aluminum^22,32,33^.

CAC forms an aluminosilicate network in combination with water glass^18^. Reaction mixtures with a Si/Al ratio of 0.1 to 0.7 were mixed from water glass (modulus 3.4), solid NaOH and CAC (CA-14 M). The mixture with the Si/Al ratio of Si/Al = 0.7 hardens after 4 h, the sample Si/Al = 0.1 hardens after 30 min.

**Fig. 3:**
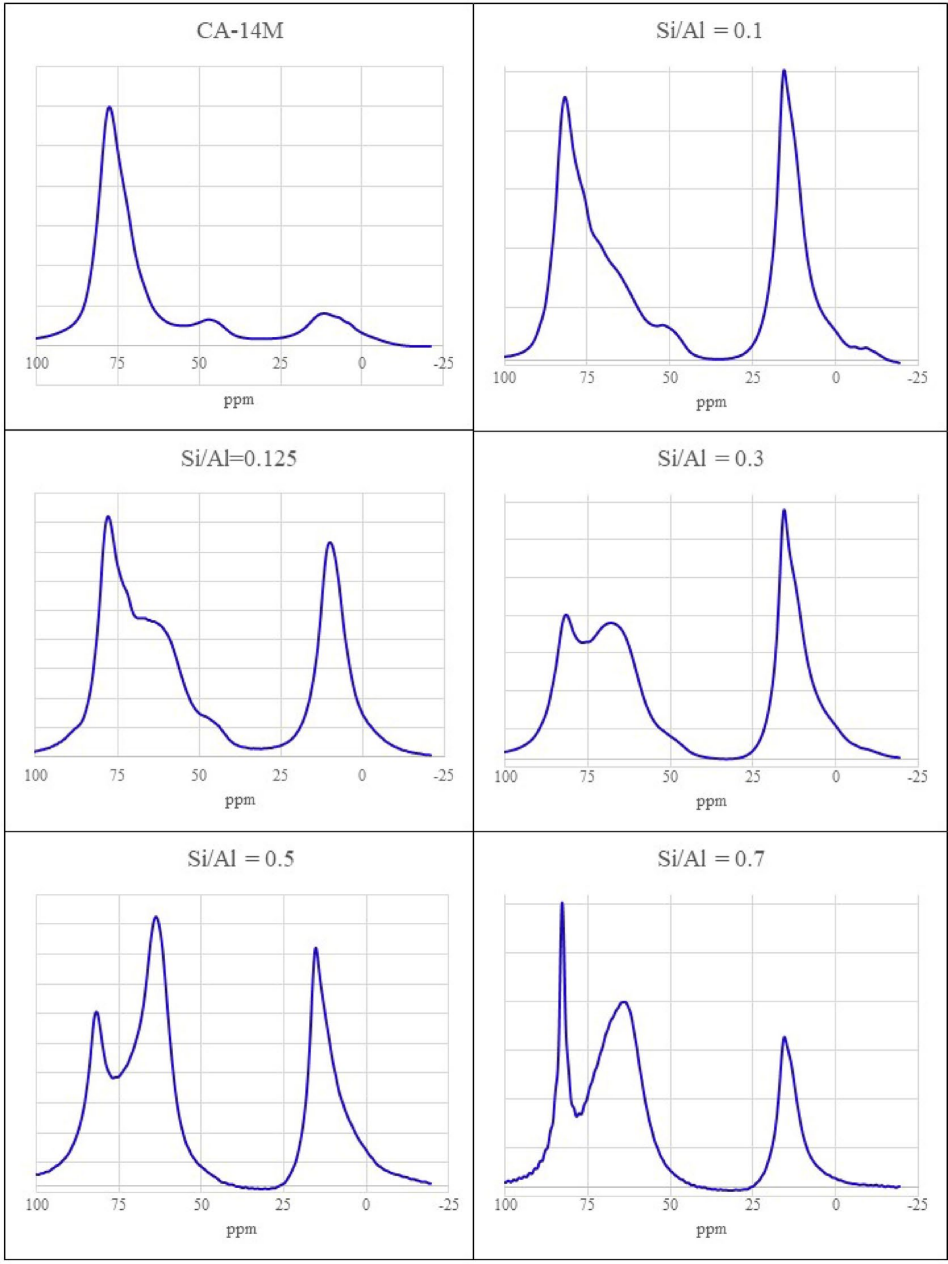
^27^Al-MAS-NMR spectrum of pure CA-14 M and spectra of different polymer compositions with Si/Al ratios from Si/Al = 0.1 to Si/Al = 0.7. The signal at 81 ppm is due to Al^IV^-coordinated aluminum. Sixfold coordinated Al-centers (Al^VI^) show signals in the range from 0-20 ppm. The broad signal around 65 ppm consists of Al^IV^ centers and is typical for an –Al–O–Si– network.

In Fig. 3, six ^27^Al-MAS-NMR spectra are plotted for Si/Al ratios of pure CA-14 M and 1/10, 1/8, 3/10, 5/10 and 7/10. The corresponding peak areas of each ^27^Al signal are listed in the supplement. The original Al^IV^ peak of CAC at 81 ppm decreases with increasing Si/Al-ratio and an additional signal appears at 65 ppm, which increases with increasing Si/Al-ratio. The signal around 65 ppm is broad because there are differently substituted Si groups in the surroundings of the tetrahedrally coordinated Al atoms. This broad signal of Al^IV^ centers is typical for an -Al-O-Si- network^8,14,18,24,25,34^. In the ^27^Al-NMR spectra with Si/Al = 0.1, Si/Al = 0.125 and Si/Al = 0.3, the peak at 47 ppm of CA_2_ is visible, indicating that this calcium aluminate did not react in these mixtures. In Si/Al = 0.5 and Si/Al = 0.7, this peak has disappeared, indicating that CA_2_ has now reacted completely. The peak at 15 ppm is typical for Al^VI^ coordinated aluminum and decreases with increasing Si/Al ratios (Fig. 3). It is probably originated from Al(OH)_3_ and indicates a partial hydration of the calcium aluminate due to the presence of water in the reaction mixture. The conversion of Al^IV^ to Al^VI^ during calcium aluminate hydration is time-dependent^22,32,35^. Despite the addition of water from water glass, a large proportion of the aluminum remains fourfold coordinated and does not immediately hydrate to sixfold coordinated aluminum. This favors the polymerization reaction under alkaline conditions over the hydration of CACs.

The Loewenstein rule states that the bonding of two Al^IV^ – tetrahedra via a common oxygen atom in zeolites is unstable, so that no more than 50% of all tetrahedra in zeolites can be Al^IV^ tetrahedra^36,37^. Quantum chemical calculations and NMR data of glasses and natural minerals emphasize the formation of –Si–O–Al– bonds compared to the formation of –Si–O–Si– and –Al–O–Al– bonds^36^. The reaction enthalpy data also favor a reaction between silicate and aluminum tetrahedra as the preferred reaction^37^. Theoretical calculations have shown that this tendency is mainly due to the exothermicity of reaction (1) both in solution and in the solid state^36,38,39^.

1 $$\equiv {\mathrm{Si}} {-} {\mathrm{O}} {-} {\mathrm{Si}} \equiv + \equiv {\mathrm{Al}} {-} {\mathrm{O}} {-} {\mathrm{Al}} \equiv \Leftrightarrow {\mathrm{2}} \equiv {\mathrm{Si}} {-} {\mathrm{O}} {-} {\mathrm{Al}} \equiv$$

All these statements support the main assumption of this work that the optimal reaction ratio of tetrahedral silicon groups and tetrahedral aluminum groups is 1:1. The reason why calcium aluminates react at room temperature is probably due to the availability of tetrahedral aluminum. Interestingly, ancient Roman concrete, used by Roman architects since the 1st century BC, contains calcium atoms and, from volcanic sources, aluminum atoms in tetrahedral (and octahedral) configuration^40–42^.

### Optimization of the Na^+^/Si ratio using setting time measurements

**Fig. 4:**
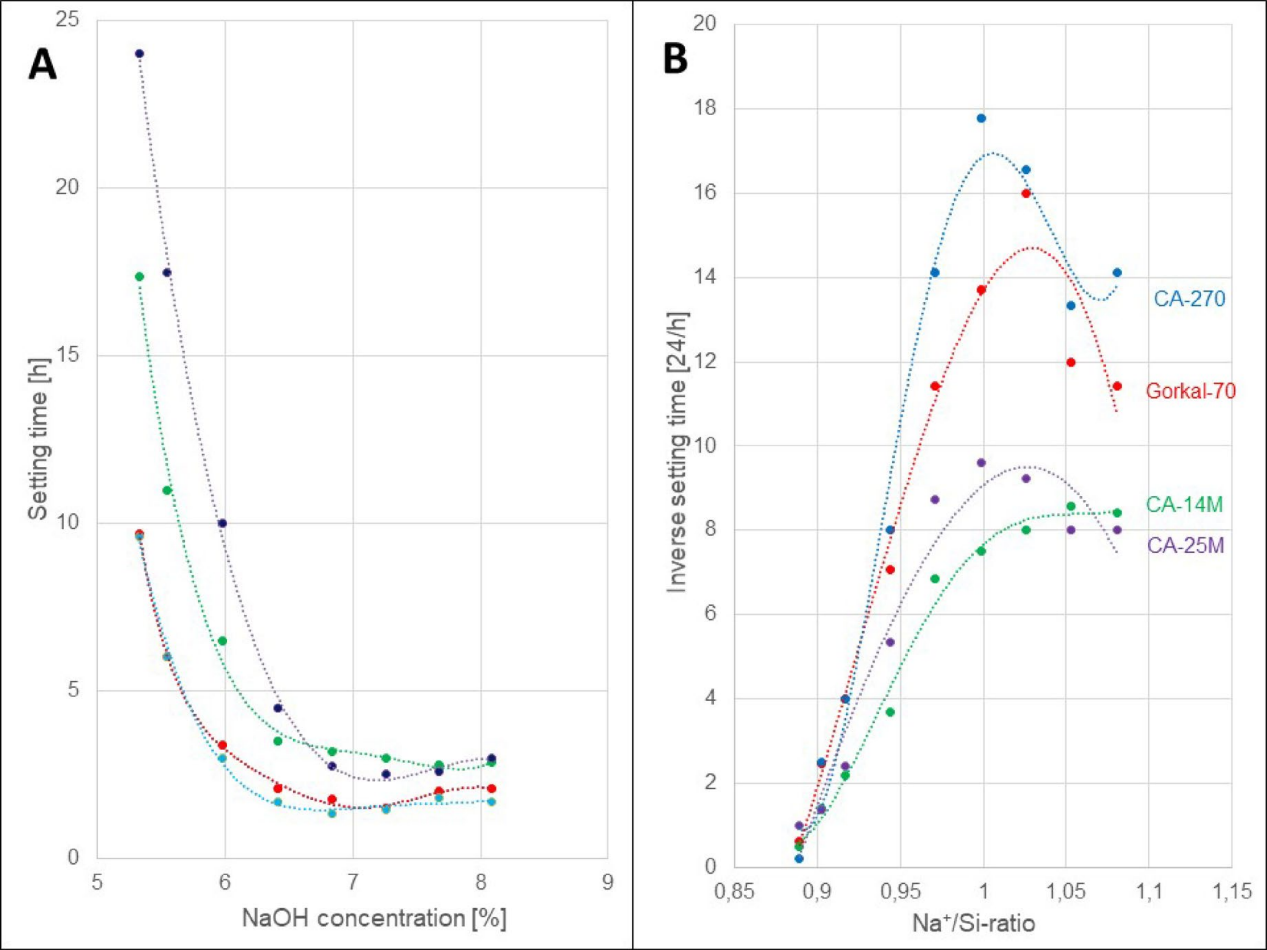
Shown is the dependence of the setting time at 20 °C (**A**) and the reverse setting time (**B**) of various CACs with different NaOH concentrations, reacted with a constant amount of water glass.

To investigate the setting time of water glass with different NaOH, different CACs with different amounts of NaOH were mixed with a constant amount of water glass (with modulus 3.4) and sand. Additional data are listed in an EXCEL-file. Figure 4A shows that the mixtures have a setting time of about 2.5 h at high NaOH concentrations (8% w/w). The optimum setting time for all CACs is at a NaOH concentration of 7.2%. At a NaOH concentration of 5.5% (w/w), the setting time was between 10 and 24 h, depending on the type of CAC. Mixtures with a NaOH concentration of slightly less than 5.1% (w/w) did not react.

Figure4B shows the relationship between the inverse setting time and the Na^+^/Si-ratio. All the curves for the different CACs have in common that no reaction takes place below a Na^+^/Si-ratio of 0.87. The reason for this is a question of the water glass composition. With increasing NaOH concentration in water glass, the sodium silicate distribution shifts towards smaller molecules, which leads to decreasing modulus values^43,44^. A Na^+^/Si-ratio of 0.87 corresponds to a water glass formula of Na_2_O*2.3SiO_2_, which means that only water glass with a modulus of *<* 2.3 reacts with CACs. The inverse setting time for all CACs has an optimum at a Na^+^/Si-ratio of 1:1 (please see Fig. 4 and^44^. This corresponds to a water glass formula of Na_2_O*2SiO_2_ and thus implies a Na^+^/Al-ration of 1:1 in the reaction.

## Discussion

Water glasses are stable liquids which do not polymerize on their own because their R-SiO^−^ groups are protected by Na^+^ or K^+^ ions. Replacement by protons at pH values < 7 initiates a condensation reaction to form Si–O–Si bonds and water. At higher pH values, the alkali cations must be actively removed to form Si–O–Si bonds, and the nature of reaction changes from condensation to polymerization^45^. The polymerization of water glass is induced by the addition of CO_2_, organic carbonates such as propylene carbonate^46^, and urea^47^, because all of these compounds bind alkali cations. This is basically the same mechanism by which calcium aluminates form silicate polymers. Here, the CACs serve as a source of negatively charged Al^IV^ centers which act as alkali cation scavengers. However, unlike the use of propylene carbonate or urea, the alkali scavenger is incorporated into the polymer structure^15^. The reaction optimum between CACs and water glass is at a sodium-to-silicate ratio of Na^+^/Si = 1:1. With a silicate-to-aluminum ratio of Si/Al = 1:1, this automatically results in a sodium-to-aluminum ratio of Na^+^/Al = 1:1. The number of positive charges that move as counterions (Na^+^ or K^+^) to the negatively charged Al^IV^ centers of the CACs is equal to the number of their reactive Al centers. This indicates that the aluminum atoms are released from the calcium aluminate framework by the formation of Na^+^Al^−^ groups. As a result, the aluminum ions become mobile and can find silicon atoms for further reactions. This also releases Ca^2+^ ions from the calcium aluminate.

In summary, the broad ^27^Al NMR peak at 65 ppm shown in Fig. 3 is consistent with Ca–Na–Al–Si–OH gels, as described in the literature for Roman concrete^12,14^ and geopolymers^6,31,34^. Due to its broad structure, this peak (in the tetrahedral region) can be interpreted as amorphous aluminum silicate, whose charge is balanced by Ca^2+^, Na^+^, or H^+^ cations in the interlayers^31^. To determine the final structure, ^29^Si-MAS-NMR and X-ray diffraction measurements are required.

### Polymer applications

The new polymeric reaction produces a suspension with low viscosity in which large quantities of additives can be dispersed. This enables the solidification of all dispersible materials such as sand, dune sand, stones, iron silicate, perlite, vermiculite and even soil and rock salt. The new polymer can also bind organic material such as wood chips, fibers, cotton and biochar. It is ideal for the production of chipboard for furniture. The most promising application is the combination of dune sand with gravel and stones for the construction of buildings. The use of uncleaned dune sand with a grain diameter of 125 μm reduces the cost of crushing stones and enables the production of bricks with a compressive strength of better than 40 N/mm^2^.

**Table 3:** Composition of one cubic meter of different polymer concretes with fine aggregate (sand < 1 mm) and coarse aggregate (2–10 mm), *with dune sand from China (125 μm), and **with plant coal (showing a negative CO_2_ emission of 320*44/12–100 = 1073 Kg CO_2_/m^3^).

Name	Na38/40 + NaOH	Al-source CA14M+CA25M	Sand, gravel	Averaged compressive strength	CO_2_-emissions (% from OPC)
A762c-d	232 Kg + 16 Kg	31.5 Kg + 96 Kg	1938 Kg	40.9 N/mm^2^	44.4 Kg CO_2_/m^3^ (32%)
A794c	285 Kg + 18 Kg	97 Kg + 0 Kg	1823 Kg	40 N/mm^2^	52.1 Kg CO_2_/m^3^ (37%)
T4, T14 (4 °C)*	514 Kg + 41 Kg	86 Kg + 209 Kg	1194 Kg	43.6 N/mm^2^	104 Kg CO_2_/m^3^ (74%)
T5, T15 (24 °C)*	483 Kg + 39 Kg	78 Kg + 196 Kg	1121 Kg	51,8 N/mm^2^	97 Kg CO_2_/m^3^ (69%)
T8, T18 (45 °C)*	489 Kg + 44 Kg	78 Kg + 199 Kg	1134 Kg	38.0 N/mm^2^	101 Kg CO_2_/m^3^ (71%)
T9, T19 (65 °C)*	473 Kg + 43 Kg	76 Kg + 192 Kg	1097 Kg	32.5 N/mm^2^	98 Kg CO_2_/m^3^(70%)
MK246CC1-2**	573 Kg + 37 Kg	195 Kg + 0 Kg	320 Kg plant coal	14.5 N/mm^2^	100 Kg CO_2_/m^3^ (71%)

Table 3 shows an example of bricks made from dune sand (T5 and T15) cast at 24 °C. The reaction can be used to produce bricks at temperatures from 4 °C to 65 °C (see Table 3, T4, T14, T5, T15, T8, T18 and T9, T19). At -21 °C, the reaction mixture freezes, but hardens immediately after thawing, even if it has been frozen for years (see T2 and T12 in additional data).

### Simplified environment assessment

The Paris Agreement aims to peak global greenhouse gas emissions as soon as possible, and this is a real problem for OPC manufacturers. There are two main sources of CO_2_ emissions in the production of OPC, the combustion of fossil fuels to generate heat and the release of CO_2_ from CaCO_3_ to produce calcium oxide (CaO). The amount of CaO in cement is called the clinker ratio and has been estimated to be 0.635 g/g^1^. Calcium oxide is the essential ingredient to form the main components in OPC, tricalcium silicate (alite) and dicalcium silicate (belite). This so-called calcination process contributes about 5% to the total anthropogenic CO_2_ emissions^1^. The combustion of fossil fuels accounts for 3% of total CO_2_ emissions worldwide 1. These emissions could be avoided if solar energy were used for the thermal treatment of CaCO_3_ for the production of OPC^1,48,49^. For example, the production of one kg of NaOH from NaCl today emits 1.915 kg CO_2_, but 96% of these emissions come from the coal used to generate electricity^50^. In a solar world, the entire energy consumption for mixing, crushing, milling or sieving can be made CO_2_-neutral by using solar power.

To produce 1 m^3^ of concrete with a compressive strength of 40 MPa (28-day strength), 240 to 640 Kg of OPC are required per m^3^ of concrete^3^. Assuming 280 kg OPC as a reasonable value, the unavoidable CO_2_ emission is 280Kg*0.635*44/56 = 140 Kg CO_2_ for 1m^3^ of concrete. The new polymer emits one mole of CO_2_ for each mole of calcium in the CACs and a single mole of CO_2_ for two moles of NaOH (made from Na_2_CO_3_) in the water glass. The optimized polymer reaction requires one mole of Na^+^ per mole of aluminum in the calcium aluminate. The total CO_2_ emission of the optimized reaction can be calculated from the CAC mass (*m*^*CAC*^) with the molar mass *M*^*CAC*^, which consists of *k*^Ca^ calcium and *k*^Al^ aluminum atoms (see Table 1), according to Eq. (2).

2 $${m^{{\mathrm{C}}{{\mathrm{O}}_2}}}=44\left( {{k^{{\mathrm{Ca}}}}+\frac{{{k^{{\mathrm{Al}}}}}}{2}} \right)\frac{{{m^{CAC}}}}{{{M^{CAC}}}}$$

A m^3^ new concrete (optimized) from 285 Kg water glass and 18 Kg NaOH (modulus 2) and 97 g CAC (CA-14 M) is responsible for 44 g/mol*(7 + 18/2)*97Kg/(1310 g/mol) = 52.1Kg CO_2_ (37% of OPC concrete). Table 3 shows the CO_2_ emissions of various CAC polymers compared to the CO_2_ emissions of an OPC concrete with an output of 140 Kg CO_2_ per m^3^. With a higher content of coarse aggregate, it is possible to produce concrete that has at least 68% lower CO_2_ emissions than OPC concrete (see samples A762c-d). The addition of biochar (produced from straw, wood and even manure by pyrolysis) to the polymer suspension results in bricks with a density of d = 1.12 g/mL, a compressive strength of 14.5 N/mm^2^ and a thermal conductivity of 0.4^−1^ K^−1^result. Such bricks can store more than a ton of CO_2_ per m^3^ of bricks (see samples MK246CC1, 2 in Table 3).

## Conclusion

The new silicon/aluminum polymer reaction requires a strongly basic environment and tetrahedrally configured aluminum in the presence of water-soluble silicates like sodium or potassium water glass. These conditions correspond exactly to those we know from Roman concrete, where silica hardens in the presence of CaO and water when volcanic glass from Neapolitan tuff containing tetrahedral aluminum is added. The main advantage of the reaction presented is that the exact stoichiometric composition of all raw materials is known and only material is required that is available worldwide in large quantities. The use of raw materials with unknown composition and doubtful future availability, such as slag or fly ash, is not necessary. In combination with aggregates like dune sand and coarser particles, the new reaction is well suited as a substitute for OPC, as it is simply carried out by mixing a solid (calcium aluminate cement and aggregates) and a liquid (alkali activated water glass solution) at room temperature. Having an OPC alternative is the first step, but it must also be accepted by the construction industry. The advantage of the polymer presented is that all equipment used in the processing of OPC can continue to be used. No special training is required for workers, as the method of concrete processing remains unchanged. By using solar energy, global CO_2_ emissions from OPC production can be reduced from 8 to 5%. The replacement of OPC concrete with water glass and CACs enables a further reduction to less than 2%. The workability of the new polymer is nearly identical to that of OPC concrete. It is important that water is present during the whole curing process of ca. 10 days. All this gives hope that the approach presented will be accepted by all those who work in today’s OPC world and see the CO_2_ problem but hesitant to stop using OPC.

## Method

Calcium aluminates ALMATIS CA-14 M, CA-270, CA-25 M and CA-25R come from Almatis GmbH, Frankfurt, Germany, and Gorkal-70 is from Gorkal, Trzebinia, Poland. Sodium hydroxid (NaOH) with 98% purity was obtained from Chem solute, Th. Geyer, Renningen, Germany. Calcium carbonate (14 μm) was from Merck, Darmstadt, Germany. The water glass Betol 38/40 (here indicated as Na38/40) from Woellner, Ludwigshafen, Germany, was used for all experiments. This water glass is a 35.8% solution of sodium silicate in water with a molar SiO_2_/Na_2_*O*-ratio of *s* = 3.4 (water glass modulus) a density of 1.37 g/cm^3^ and a viscosity of 100 mPas. Its pH-value (100 g/L) is 11.3. γ-Al_2_O_3_ was purchased from Nabaltec AG, Schwandorf, Germany. SIRKON^®^ quartz (quartz powder) was purchased from Quarzwerke Frechen, Frechen, Germany and has an average grain size of 8 μm. Dune sand was collected in China and has an average grain size of 125 μm (70–200 μm) and was used without any pre-treatment or cleaning steps. Construction sand (0.18–1 mm) was obtained from a local purchaser, as was granite-gravel (crushed stone) in a 0–1.5 cm size. The mixed sand was proportioned according to *Fuller* and *Thompson*^23^ to improve the packing density of the concrete (Fuller-sand). To 650 g of construction sand, 120 g of dune sand from China and 30 g of quartz powder or calcium carbonate were added. Polystyrene cubes (4 × 4 × 4 cm^3^) as casting molds (finger food trays square no. 10524) and lids (finger food plates square no. 11208) were supplied by PAPSTAR^®^ GmbH, Kall, Germany.

### General procedure

Activated water glass was prepared by dissolving 6–10 g of NaOH in 100 g of water glass by simple shaking. The calculated amount of calcium aluminate was mixed with the clear solution to form a white suspension, which has its lowest viscosity after 10 min mixing. The sand and stones were then added by mixing. The suspension was cast in 4 × 4 × 4 cm^3^ polystyrene cubes.

Mixing activated water glass with CACs results in a fluid suspension to which sand and other additives can be easily added to produce a pourable medium viscose paste. To study different CACs, the amount of 8.8 g NaOH was dissolved in 91.2 g water glass (Betol 38/40). The solution was mixed with each of the CACs Gorkal-70, CA-14 M and CA-270 M for 5 min, and then 280 g sand (0–1 mm) was added. For CA-25 M, 50 mL of a 8.8% NaOH in water glass was mixed with around 340 g of sand mixture (0–1 mm sand and dune sand). Cubes were cast from these mixtures, which were cured at room temperature for 7 days in a mold closed with a lid, then removed and dried for three days. A sheet of polyethylene was placed between the cube and the lid to seal the sample against CO_2_ from air.

To study the reaction kinetics, different amounts of NaOH (5.0–9.0 g) were dissolved in 100 g of water glass Na38/40. Different amounts of CACs were added to each solution in the ranges CA-14 M (28.7–36 g), Gorkal-70 (29.7–37.2 g), CA-270 M (38.2–47.9 g) and CA-25 M (47.8–59.9 g). After stirring for 5 min, the amount of 120 g sand was added. The mixtures were then filled into flexible polystyrene cubes (4 × 4 × 4 cm^3^). The initial setting time was checked by squeezing the plastic cubes with two fingers at 15-minute intervals to determine whether the mixture was liquid or stiff.

### NMR-measurements

Solid-state ^27^Al NMR spectra were recorded from fine polymer powder using a Brucker Avance II 400 MHz Solid State-NMR spectrometer (9.4 T) operated at 104.19 MHz for ^27^Al. A commercially available double-resonance MAS probe (4 mm) was used and the spinning speed was 10 kHz for all the spectra with accuracies of (2 Hz). The spectra were recorded with a single pulse excitation using a π/6 pulse of 1 µs pulse length and a 2s recycle delay. The spectra were referenced to 1 M aqueous solution of AlCl_3_–6H_2_O as external standard (0 ppm). Solid cryolite (Na_3_AlF_6_) was used as a secondary reference. Data processing was performed with the program TOPSPIN3.5 (Bruker). Peak integration was performed by summing the signal intensities.

^29^Si NMR spectra were measured on a Brucker Advance 500 DSX 500WB at room temperature with 10 kHz spinning speed and a single pulse excitation using a π/4 pulse of 2 µs pulse length and 6s recycle delay. The spectra were referenced using a silicon Q_8_M_8_ standard.

### Infrared measurements

For Fourier-transform-infrared (FTIR)-measurements, a Nicolet iS5 with ATR-unit iD5-diamond from Thermo Scientific GmbH, Dreieich, Germany, was used. The reaction mixture was poured onto the diamond and recorded as a function of time in a range from 400 to 4000 cm^–1^. Each spectrum was measured in absorbance and averaged from nine single spectra, measured with a resolution of 0.8 cm^–1^.

### Compressive strength measurements

Compressive strength measurements were performed in duplicate on 40 × 40 × 30 mm samples using a Zwick/Roell Z250 from Zwick GmbH, Ulm, Germany.

## Electronic Supplementary Material

Below is the link to the electronic supplementary material.

Supplementary Material 1

## Data Availability

This article contains original data in an EXCEL file which are available as: Additional data to Calcium aluminate and activated water glass as an eco-friendly inorganic polymer.XLSX.
